# The Budget and the Doctor's Car

**Published:** 1909-05-29

**Authors:** 


### Motoring Notes.

### THE BUDGET AND THE DOCTOR'S CAR.

### The New Taxes on Cars and Petrol.

The proposals of the Chancellor of the Exchequer in regard to new taxes on motor cars and petrol are, no doubt, already familiar to readers. The new taxes on cars do not come into force until next January, but the petrol tax is already making its influence felt. During the last few years public speakers, who have voiced the views of some of the leading motor organisations, have constantly urged that they would not object to a revision of the motor-car licences, provided it was accompanied by the creation of a central department responsible for the maintenance of the existing roads and the construction of new ones, designed with full regard to the exigencies of the new traffic.

Mr. Lloyd George has not only taken these advocates at their word, but has gone beyond their well-meant suggestions by a higher scale of taxation for cars, and also by adding 30 per cent. to the cost of running them on the road. The tax on petrol is altogether out of proportion to the necessities of the case, and it is generally believed that the Chancellor's estimate of the revenue likely to accrue will probably be found an under-statement of the position. Had the proposal been for a 2d. tax with a rebate of half in the case of cars used for commercial or professional purposes, the imposition could be borne with more resignation; but to increase the price by more than 30 per cent. is really unwarranted. As doubtless my readers know, this imposition of 3d. per gallon has already led to an increase of 4d. to the user. The extra penny has been added by the petrol companies to cover the cost of systems of checking, granting certificates, claims for exemption, etc., and other detailed work which necessarily involves the importers in additional expense. There has been a certain amount of discussion as to whether doctors are eligible for the petrol rebate, and it undoubtedly seems anomalous that they should receive a rebate on the tax of the car and not on that of the petrol that drives the car. I regret to say that the Chancellor has lately stated that medical men will not receive this favour. After all, we have something to be thankful for in regard to the rebate on the car tax, and we must not forget that we are the only class of professional man whose claims to exceptional treatment have been recognised. I understand that representations have been made to the Chancellor by veterinary surgeons, school inspectors, etc., for a similar rebate, but without effect.

### Taxation by Cylinder Bore.

With regard to the classification of motor cars for the purposes of taxation, this has hitherto been based on the weight of the car, but at the present time it is very generally agreed that power rather than weight should be the basis of graduation. Taxation by cylinder bore, as proposed, appears to be the only way in which the horse-power may be readily arrived at, and it is fairly accurate for ordinary touring cars. There is little doubt that the Royal Automobile Club's formula will be used for rating purposes. In this

$$\frac{D^2 \times N}{2{\cdot}5} = \text{H.P.}$$

D being the diameter of the cylinder in inches, and N the number of cylinders.

The following table gives the bore (in inches and millimetres) and the R.A.C. horse-power of most of the 1, 2, and 4-cylinder cars that are used by medical men. By reference to this anyone knowing the bore of his cylinder or cylinders can see at a glance the horse-power of his car and the licence fee that will in future have to be paid, not forgetting, of course, that half-rates only in each class are to be paid by members of the profession.

**Single-cylinder Cars.**

| Licence £2 2s. per annum | | | Licence £3 3s. per annum | | |
|---|---|---|---|---|---|
| Bore | | | Bore | | |
| Inches | m.m. | H.P. | Inches | m.m. | H.P. |
| $3\frac{1}{2}$ | 89 | 4.9 | $4\frac{1}{8}$ | 105 | 6.8 |
| $3\frac{5}{8}$ | 92 | 5.25 | $4\frac{1}{4}$ | 108 | 7.2 |
| $3\frac{3}{4}$ | 95 | 5.6 | $4\frac{3}{8}$ | 111 | 7.6 |
| $3\frac{7}{8}$ | 98 | 6 | $4\frac{1}{2}$ | 114 | 8.1 |
| $3\frac{15}{16}$ | 100 | 6.2 | $4\frac{5}{8}$ | 117 | 8.5 |
| 4 | 102 | 6.4 | $4\frac{3}{4}$ | 124 | 9 |
| | | | $4\frac{7}{8}$ | 124 | 9.5 |
| | | | 5 | 127 | 10 |

**Two-cylinder Cars.**

| Licence £3 3s. per annum | | | Licence £4 4s. per annum | | |
|---|---|---|---|---|---|
| Bore | | | Bore | | |
| Inches | m.m. | H.P. | Inches | m.m. | H.P. |
| $2\frac{15}{16}$ | 75 | 6.9 | $3\frac{7}{8}$ | 98 | 12 |
| 3 | 76 | 7.2 | $3\frac{15}{16}$ | 100 | 12.4 |
| $3\frac{1}{8}$ | 79 | 7.8 | 4 | 102 | 12.8 |
| $3\frac{5}{32}$ | 80 | 7.9 | $4\frac{1}{8}$ | 105 | 13.6 |
| $3\frac{1}{4}$ | 83 | 8.4 | $4\frac{1}{4}$ | 108 | 14.4 |
| $3\frac{3}{8}$ | 86 | 9.1 | $4\frac{3}{8}$ | 121 | 15.3 |
| $3\frac{1}{2}$ | 89 | 9.8 | | | |
| $3\frac{5}{8}$ | 92 | 10.5 | | | |

**Four-cylinder Cars.**

| Licence £3 3s. per annum | | | Licence £4 4s. per annum | | |
|---|---|---|---|---|---|
| Bore | | | Bore | | |
| Inches | m.m. | H.P. | Inches | m.m. | H.P. |
| $2\frac{11}{25}$ | 62 | 9.4 | $2\frac{3}{4}$ | 70 | 12.4 |
| $2\frac{1}{2}$ | 64 | 10 | $2\frac{7}{8}$ | 73 | 13.2 |
| $2\frac{14}{25}$ | 65 | 10.6 | 3 | 76 | 14.4 |
| $2\frac{5}{8}$ | 67 | 11 | $3\frac{1}{8}$ | 79 | 15.6 |

It is unnecessary to work out the R.A.C. horse-power of the 3-cylinder type of engine, as this variety is practically obsolete, and at the present time is used by only one or two manufacturers, but most medical men will find their car's cylinder dimensions in the table.

As stated above, the Chancellor the Exchequer, when recently questioned with regard to the eligibility of medical men for the rebate in the petrol tax, replied in the negative. It appears, however, that there is some hope of concession after all, as since then, replying to a further question, he intimated his willingness to receive a deputation on the matter if he could spare the time. It is to be hoped that he can, since if so it should not be difficult to convince him of the very great hardship of this duty, which, as a member of the House stated, practically amounts to a special income-tax on the medical profession. "Viator."

